# Composite Tissue-Engineered Small-Diameter Vascular Grafts Based on Polycaprolactone and Polyurethane with Growth Factors and Atrombogenic Drug Coatings: Surface Ultrastructure, Physical and Mechanical Properties

**DOI:** 10.17691/stm2024.16.5.02

**Published:** 2024-10-30

**Authors:** E.A. Senokosova, E.S. Prokudina, E.S. Krivkina, T.V. Glushkova, E.A. Velikanova, M.Yu. Khanova, E.A. Torgunakova, V.G. Matveeva, L.V. Antonova

**Affiliations:** PhD, Researcher, Laboratory of Cell Technologies; Research Institute for Complex Issues of Cardiovascular Diseases, 6 Academician L.S. Barbarash Blvd, Kemerovo, 650002, Russia; MD, PhD, Researcher, Laboratory of Tissue Engineering and Intravascular Imaging; Research Institute for Complex Issues of Cardiovascular Diseases, 6 Academician L.S. Barbarash Blvd, Kemerovo, 650002, Russia; Junior Researcher, Laboratory of Cell Technologies; Research Institute for Complex Issues of Cardiovascular Diseases, 6 Academician L.S. Barbarash Blvd, Kemerovo, 650002, Russia; PhD, Senior Researcher, Laboratory of New Biomaterials; Research Institute for Complex Issues of Cardiovascular Diseases, 6 Academician L.S. Barbarash Blvd, Kemerovo, 650002, Russia; PhD, Researcher, Laboratory of Cell Technologies; Research Institute for Complex Issues of Cardiovascular Diseases, 6 Academician L.S. Barbarash Blvd, Kemerovo, 650002, Russia; Junior Researcher, Laboratory of Cell Technologies; Research Institute for Complex Issues of Cardiovascular Diseases, 6 Academician L.S. Barbarash Blvd, Kemerovo, 650002, Russia; Research Assistant, Laboratory of Cell Technologies; Research Institute for Complex Issues of Cardiovascular Diseases, 6 Academician L.S. Barbarash Blvd, Kemerovo, 650002, Russia; MD, PhD, Senior Researcher, Laboratory of Cell Technologies; Research Institute for Complex Issues of Cardiovascular Diseases, 6 Academician L.S. Barbarash Blvd, Kemerovo, 650002, Russia; MD, DSc, Head of the Laboratory of Cell Technologies; Research Institute for Complex Issues of Cardiovascular Diseases, 6 Academician L.S. Barbarash Blvd, Kemerovo, 650002, Russia

**Keywords:** tissue-engineered vascular prosthesis, polyurethane, polycaprolactone, electrospinning

## Abstract

There is a growing need for synthetic small-diameter vascular grafts (<6 mm) for bypass surgery since the majority of currently developed products have demonstrated unacceptable high frequency of thrombosis in preclinical studies. The proprietary composite vascular graft based on a nonwoven polymer with anti-thrombogenic and anti-aneurysm effect and functional activity is aimed at stimulating the formation of vascular neotissue at the implantation site.

**The aim of the investigation** is to study the surface morphology, physical and mechanical properties of the polycaprolactone/ polyurethane (PCL/PU) small-diameter tissue-engineered vascular graft with growth factors and an anti-thrombogenic surface coating.

**Materials and Methods:**

PCL/PU vascular grafts with growth factor mix (GFmix) were manufactured using the electrospinning method. The hydrogel coating containing iloprost (Ilo) and heparin (Hep) was formed by complexation with polyvinylpyrrolidone. The controls were multilayer vascular grafts of similar composition and nonwoven matrices based on 12% PCL and 12% PU. The surface structure was analyzed with the S-3400N scanning electron microscope (Hitachi, Japan). The physical properties of the surface were determined by the wetting angle method. The mechanical properties were evaluated on a Z series universal testing machine (Zwick/ Roell, Germany). Statistical processing of the data was performed using the GraphPad Prism 8 software.

**Results:**

Our new manufacturing technique for the composite PU/PCL/GFmix/^Ilo/Hep^ graft has eliminated the problem of graft delamination. The inner surface of the graft consisted of interwined microfibers (1.34 [1.15; 2.28] μm thick), nanofibers (790.0 [604.0; 853.5] nm thick), and interpenetrating pores of different diameters (5.4 [3.8; 8.4] μm). The process of coating formation did not affect the fibers and did not seal the pores, the surface retained its hydrophilic properties (θ=68.61±11.85°). The tensile strength (3.45 [3.17; 4.03] MPa) and Young’s modulus (4.88 [3.95; 5.80] MPa) of PU/PCL/GFmix/^Ilo/Hep^ grafts were almost similar to the human internal thoracic artery compared to the multilayer analogs. The PU/PCL/GFmix/^Ilo/Hep^ grafts were superior to the multilayer PCL/PU/GFmix/^Ilo/Hep^ grafts in terms of reduced excessive elasticity (to 118.0 [111.0; 125.0]%; p=0.043).

**Conclusion:**

The composite functionalized vascular PU/PCL/GFmix/^Ilo/Hep^ grafts have enhanced characteristics and compliance, which, in turn, increases the probability of their high patency in future preclinical studies.

## Introduction

Vascular diseases are one of the leading causes of disability and mortality in the world. The standard treatment for severe vascular diseases is bypass surgery using autologous arteries or veins, however, their application is limited due to a number of factors [1–3]. The main synthetic materials for vascular reconstruction of blood vessels such as Gortex, Dacrone, and ePTFE (d>6 m) are prone to neointimal hyperplasia, calcification and bacterial infection resulting in the reduction of patency of these grafts and a high risk of complete incompetence in the long-term period [4–6]. These materials are entirely unsuitable as substitutes for small-diameter vessels (d<6 mm) because of their rapid thrombosis [7–9]. Some natural commercial grafts such as Artegraft®, ProCol®, and Omniflow II® represent biosynthetic prostheses based on bovine vessels and are used as a shunt for dialysis and replacement of large-diameter vessels. These alternatives also have key limitations preventing their application in the bypass surgery on the small-caliber vessels: inconsistency of mechanical properties, the risk of aneurysm formation and/or thrombosis, allergic reactions, and rejections [10, 11].

Presently, no effective synthetic small-diameter vascular prostheses have been created for clinical applications. A tissue-engineered vascular graft fabricated using new methods of material sciences, engineering and cell biology seems to be a promising candidate. Its advantages over autografts include non-invasive surgical intervention during graft preparation, unlimited availability, and the possibility to use an individualized approach to the graft size. However, the existing developments are far from being satisfactory. Since there are a variety of unsolved questions, searching for the materials for small-diameter vascular grafts is going on [12–15]. The majority of candidates for these prostheses are manufactured by the electrospinning technique or 3D printing from a polymer solution, which makes it possible to create a fibrous porous non-woven material similar to the natural extracellular matrix. A low blood flow rate in small-caliber vessels is responsible for a high risk of thrombosis, which the researchers face at the stage of testing their items on a large animal model [16]. To overcome this and some other problems, tissue-engineered grafts may be made functionally active: for example, by stimulating endothelialization; attracting the cells synthetizing extracellular matrix components; increasing their anti-microbial and anti-thrombotic potential; it is also possible to enhance the scaffold to prevent aneurysm formation [17–19].

Our new technology for creation a functionalized vascular prosthesis has some specific features. The first is a composite base from polycaprolactone (PCL) and polyurethane (PU). PCL possesses a sufficiently high strength and elasticity and is subject to biodegradation during 2–3 years. At the same time, gradual bioresorption of PCL will provide the possibility of full graft remodeling by replacing the polymer tubular scaffold with the patient’s own cells and tissues [20]. The presence of PCL in the scaffold composition promotes the adaptive growth of the graft upon its implantation into the vascular bed. PU is a synthetic polymer with a high biocompatibility and excellent mechanical properties, which possesses increased stability in the biological systems and is used in the biomedical industry. Incorporation of this polymer into the composition of the polymer graft scaffold will bring the physical and mechanical properties of the graft closer to the properties of the native small-diameter arterial vessels and provide the resistance of its walls to aneurysmal dilatation due to extremely low rate of hydrolytic degradation [21–23]. The combination of these polymers will impart high strength and durability, elasticity and bending resistance to the vascular graft. After the implantation of this graft into the vascular bed, formation of graft wall aneurysms in the process of its functioning in the vascular bed will be prevented preserving the possibility of full remodeling.

The second feature consists in the stimulation of the graft remodeling process after the implantation into the vascular bed with angiogenic factors: vascular endothelial growth factor (VEGF), basic fibroblast growth factor (bFGF), and chemoattractant molecules — stromal cell-derived factor 1 alfa (SDF-1α). VEGF triggers endothelialization by activating migration, proliferation, and differentiation of endothelial cells. bFGF stimulates migration, proliferation, and survival of endothelial and smooth muscle cells. SDF-1α facilitates the attraction of bone marrow-derived progenitor cells from the bloodstream to the zone of the graft location [24].

The third feature involves superficial modification of the grafts with athrombogenic drugs (iloprost (Ilo) and heparin (Hep)) to prevent lumen thrombosis of the implanted vascular prostheses in the early postoperative period [25].

The quality of the material should be controlled at each stage of tissue-engineered vascular graft fabrication, since the additional modifying procedures may change its initial characteristics. This article presents data of the *in vitro* comparison of the new technology of fabricating a monolayer functionalized composite vascular graft with the previous multilayer graft design based on the same composition.

**The aim of the study** is to evaluate the surface structure, physical and mechanical characteristics of the composite tissue-engineered vascular graft of a small diameter based on polycaprolactone and polyurethane with growth factors and antithrombogenic coating, and to compare it with the multilayer analog.

## Materials and Methods

### Fabrication of PCL and PU scaffolds

Scaffolds were fabricated by the electrospinning technique (Nanon-01A; MECC, Japan) from the solution of 12% PCL (Sigma-Aldrich, USA) and 12% PU (Tecofex EG-80A; Lubrizol Advanced Materials, USA) in chloroform using the following parameters: needle — 22 G, voltage — 20 kV, solution feed rate — 0.5 ml/h, collector rotation speed — 200 rpm, needle cleaning time — 30 s.

### Fabrication of multilayer vascular PCL/GFmix/ PU/^Ilo/Hep^ (d=4 mm) grafts

At the first stage, the grafts were manufactured in layers using the electrospinning technique from the polymer solution in chloroform (Vekton, Russia). The first (internal) layer consisted of 12% PCL + 1% Plu (Sigma-Aldrich, USA) with incorporation of VEGF (SaiStorLab, Russia), the second layer — 12% PCL + 1% Plu (Sigma-Aldrich, USA) with incorporation of bFGF (SaiStorLab, Russia) and SDF-1α (Cloud-Clone Corp., USA). The parameters used were as follows: needle — 22 G, voltage — 22 kV, collector rotation speed — 200 rpm, solution feed rate — 0.5 ml/h, needle cleaning time — 30 s, distance from the needle to the winding collector — 15 cm. The third layer contained 12% PU in chloroform. The parameters were the same except the voltage, which was 20 kV.

At the second stage, a drug coating with Ilo and Hep was formed by complexation with polyvinylpyrrolidone [26].

### Fabrication of composite monolayer vascular PCL/PU/GFmix/^Ilo/Hep^ (d=4 mm) grafts

At the first stage, the grafts were fabricated using the electrospinning technique from the polymer solution in chloroform — 8% PCL + 5% PU + 1% Plu with a concomitant introduction of the growth factor complex (GFmix: VEGF, bFGF, and SDF-1α). The following parameters of electrospinning were used: voltage — 22 kV, collector rotation speed — 200 rpm, solution feed rate — 0.5 ml/h, needle cleaning time — 30 s, distance from needle to winding collector — 15 cm.

The second stage is completely identical to the one described above.

### Surface ultrastructure

The structure of the layer surface was assessed using the S-3400N SEM (Hitachi, Japan) under a high vacuum at the 10 kV accelerating voltage. Prior to the test, a 15 nm gold-palladium coating was deposited (EM ACE200; Leica Mikrosysteme GmbH, Austria) on the 0.5×0.5 cm graft samples.

### Physical and mechanical properties

To evaluate hydrophilic/hydrophobic properties, a wetting angle on the polymer materials was determined using the sitting drop method and the Drop Shape Analyzer DSA25 (KRÜSS GmbH, Germany) at room temperature. The contact wetting angle was calculated from 5 images in the ImageJ software (NIH, USA) for each type of the polymer material.

The mechanical properties of the material were tested on the universal series Z testing machine (Zwick/Roell, Germany) using the probe with a nominal force of 50 H and an acceptable error threshold ±1%, crossbeam moving speed during testing was 50 mm/min. The material tensile strength was determined as ultimate stress in tension (MPa) prior to destruction. Since the tested biological samples and grafts differed essentially in thickness and, consequently, a cross-section area, absolute value of the maximal force applied to the sample before the beginning of destruction (Fmax, H) was used as an alternative criterion of strength. Elastic deformation of the material was assessed by relative elongation prior to sample destruction (%) and the Young’s modulus (MPa), which was determined in the range of physiological pressure (80–120 mm Hg). The mechanical properties of the grafts were compared with those of the human internal thoracic artery.

### Statistical data processing

The results were statistically processed using GraphPad Prism 8 program (GraphPad Software, USA). The character of data distribution in the samples was evaluated by Kolmogorov–Smirnov and Shapiro–Wilk tests. The qualitative data were presented as mean ± σ or median and quartile range (Me [25%; 75%]). Statistically significant differences between independent groups were assessed using the Kruskal–Wallis test with the result correction considering multiple comparisons by the FDR method. The differences were considered significant at p<0.05 for all tests.

## Results

### The structure of the material surface

**Figure 1.**
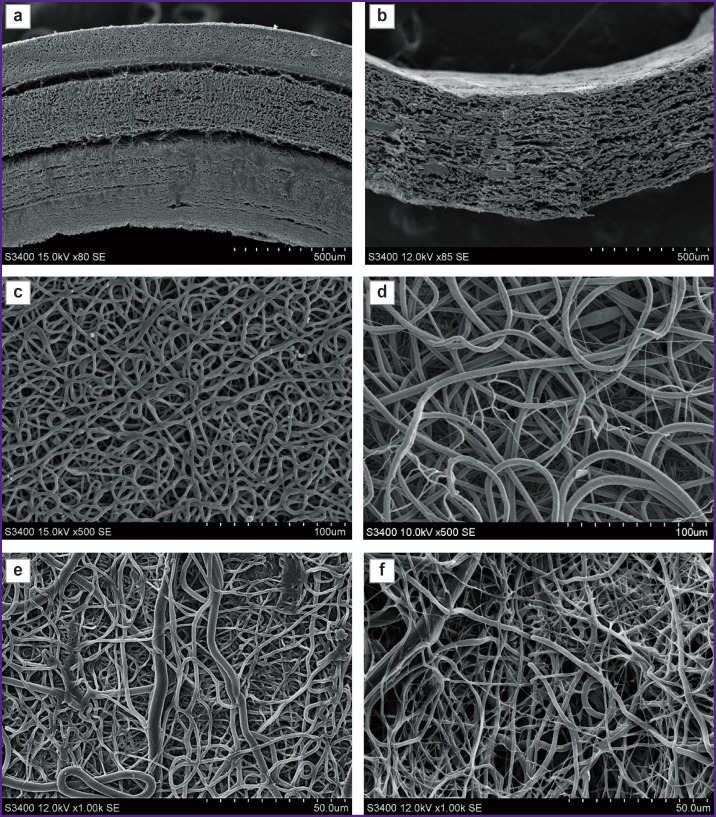
Scanning electron microscopy of the internal surface of matrices with different polymer compositions Matrix cross-section: (a) PCL/GFmix/PU/^Ilo/Hep^; (b) PU/PCL/GFmix/^Ilo/Hep^; matrix surface structure: (c) PU; (d) PCL; (e) PU/PCL/GFmix; (f) PU/PCL/GFmix/^Ilo/Hep^

**Figure 2.**
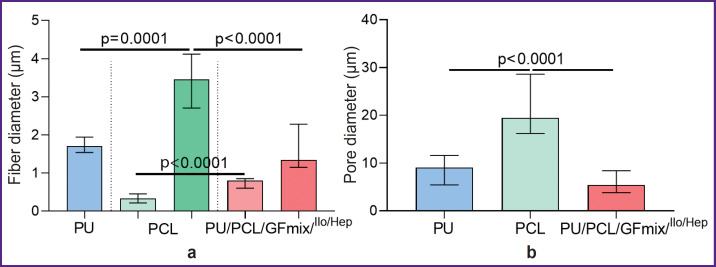
Morphological characteristics of the matrix internal surface: (a) fiber diameter; (b) pore diameter

The initial variant of fabricating a multilayer vascular graft with external PU reinforcement by the electrospinning method was characterized by the separation of the external PU layer and entire radial delamination of the PU/GFmix base due to the stepwise formation of 1/3 of the item with VEGF incorporation and 2/3 with bFGF + SDF-1α (Figure 1 (a)). The idea of combining PU, PCL and three differentiation factors in a single solution enabled us to create a tissue-engineered prosthesis without delamination of its wall (Figure 1 (b)). The PU/PCL/GFmix scaffold possessed the characteristics inherent to each monocomponent material: the internal surface consisted of interwined microfibers (1.34 [1.15; 2.28] μm thick) and nanofibers (790.0 [604.0; 853.5] nm thick) of convoluted disordered orientation with pores of different diameters (5.4 [3.8; 8.4] μm) partly compacted by the networks of tightly interwoven nanofibers. The addition of PU to PCL resulted in a statistically significant 3.6-fold reduction of the pore diameter (p<0.0001) relative to the PCL surface (19.5 [16.2; 28.6] μm). Porosity of PCL/PU/ GFmix wall was not less than 50% (Figure 1 (c)–(e); Figure 2). The PVP cross-linking with the PCL ester groups under the conditions of the argon atmosphere and gamma-radiation and further processing by the drug mix with drying did not affect the fiber structure and did not seal the pores (Figure 1 (f)).

### Physical characteristics of the material surface

**Figure 3.**
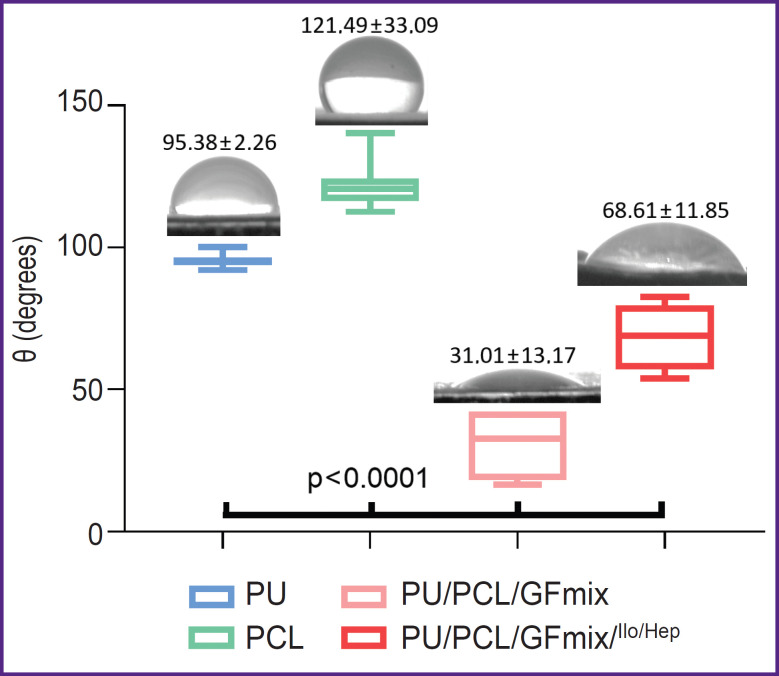
Contact wetting angle on the surface of the polymeric materials

Determination of the contact wetting angle has revealed an essential increase in the hydrophilic properties of the composite PU/PCL/GFmix material (θ=31.01±13.17°) in comparison with monocomponent analogs (p<0.0001). Formation of the drug coating promoted statistically significant 2.2-fold reduction of hydrophilic properties (p<0.0001); an average angle of contact with water was equal to 68.61±11.85° (Figure 3), however, this modified surface may be considered sufficiently hydrophilic.

### Mechanical characteristics of the composite vascular grafts

The drug coating on the PU/PCL/ GFmix grafts is known to cause no significant effect on the mechanical characteristics of the item. We managed to bring the parameters of strain and stiffness of the PU/PCL/GFmix/^Ilo/Hep^ closer to those of the human internal thoracic artery as compared to the PCL/GFmix/ PU/^Ilo/Hep^ grafts, in which polyurethane acted as an external reinforcement layer. It should be also noted that the composite PU/PCL/GFmix/^Ilo/Hep^ grafts had statistically significantly lower excessive elasticity (up to 118.0 [111.0; 125.0]%) relative to the value for the PCL/ GFmix/PU/^Ilo/Hep^ grafts (p=0.043) (see the Table).

**Table:** Mechanical properties of the composite vascular grafts, Me [25%; 75%]

Samples	Strain (MPa)	Relative elongation (%)	Young’s modulus (MPa)
Human internal thoracic artery	2.48 [1.36; 3.25]	29.72 [23.51; 39.62]	2.42 [1.87; 3.19]
PCL/GFmix/PU/^Ilo/Hep^	3.78 [3.04; 5.99]*	143.60 [120.30; 166.90]*	4.68 [3.93; 5.23]*
PU/PCL/GFmix	3.65 [2.43; 4.75]*	120.0 [104.40; 134.0]*^+^	3.32 [3.07; 3.95]*
PU/PCL/GFmix/^Ilo/Hep^	3.45 [3.17; 4.03]*	118.0 [111.10; 125.0]*^+^	4.88 [3.95; 5.80]*

* p<0.05 as opposed to the internal thoracic artery values;

^+^ p<0.05 as opposed to the PCL/ GFmix/^Ilo/Hep^/PU values.

## Discussion

To create tissue-engineered small-diameter vascular prostheses, numerous factors should be taken into consideration in order to achieve their effective functioning in the patient’s vascular bed. The graft must possess biomechanical characteristics as close to the native artery as possible for the appropriate compliance in the hemodynamic conditions and prevention of neointimal hyperplasia. This effect may be achieved by regulation of the electrospinning parameters or other methods used to fabricate non-woven materials. The current trend of developing vascular grafts is to enhance their functional activity, which is directed to solving the main problems holistically connected with effective patency in the long-term period and minimal need for graft replacement. In case of a low blood flow and increased resistance [27], there is a high risk of early graft thrombosis in this implantation projection, therefore, researchers modify them by various methods: reducing pores in the internal surface and creating antithrombotic layers, which may cause spontaneous endothelialization, or releasing locally drugs blocking thrombocyte attachment and thrombus formation. Concurrently with the problem of patency, the task of harmonization of the artificial scaffold remodeling also needs solving. This can be achieved by attracting a number of cells such as endothelial cells and fibroblasts to the surface and thickness of the graft wall. Implantation of tissue-engineered grafts into the vascular bed of the small and large laboratory animals makes it possible to evaluate de novo generated tissue consisting as a rule of the structures similar to the native vascular tissue: neointima, neomedia, neoadventitia, and fragments of the polymer scaffold [28]. This tissue is susceptible to aneurysm formation, which is what we observed in our experiments on the sheep model [29]. Reinforcement of the vascular graft scaffold making it resistant to bioresorption is also an important task in creating medical items of this class.

The proprietary design of the functionalized unwoven small-diameter vascular prosthesis based on polycaprolactone and polyurethane with the incorporated growth factor mix (VEGF, bFGF, SDF-1α) allowed us to obtain a graft with a highly porous structure without delamination of its wall. Formation of a hydrophilic hydrogel coating (θ=68.61±11.85°) using radiation polymerization of PVP with further complexation with Ilo and Hep did not affect the mechanical properties of the final product. The mechanical characteristics of the composite PU/PCL/GFmix/^Ilo/Hep^ grafts were closer to those of the native artery than the parameters of the multilayer PCL/GFmix/PU/^Ilo/Hep^ grafts and our previous designs [30] owing to the improved manufacturing technology: replacement of the polymeric component, namely, polyhydroxybutyrate/valerate with polyurethane; usage of the mix in a single polymer solution with the addition of emulsion stabilizer Pluronic instead of layered incorporation of the growth factors. This technology enabled us to create a new promising small-diameter vascular graft.

## Conclusion

The composite functionalized vascular PU/PCL/ GFmix/^Ilo/Hep^ graft possesses improved characteristics and compliance, which, in turn, may increase the probability of high patency when implanted to the large laboratory animals in preclinical trials.
